# Characterization of an Alginate Lyase, FlAlyA, from *Flavobacterium* sp. Strain UMI-01 and Its Expression in *Escherichia coli*

**DOI:** 10.3390/md12084693

**Published:** 2014-08-22

**Authors:** Akira Inoue, Kohei Takadono, Ryuji Nishiyama, Kenji Tajima, Takanori Kobayashi, Takao Ojima

**Affiliations:** 1 Laboratory of Marine Biotechnology and Microbiology, Faculty of Fisheries Sciences, Hokkaido University, Hakodate, Hokkaido 041-8611, Japan; E-Mails: inouea21@fish.hokudai.ac.jp (A.I.); k.takadono@gmail.com (K.T.); nsym2480@ec.hokudai.ac.jp (R.N.); 2 Laboratory of Molecular Materials Chemistry, Faculty of Engineering, Hokkaido University, Sapporo, Hokkaido 060-8626, Japan; E-Mail: ktajima@eng.hokudai.ac.jp; 3 Hokkaido Industrial Technology Center, Kikyou, Hakodate, Hokkaido 041-0801, Japan; E-Mail: kobayashi@techakodate.or.jp

**Keywords:** alginate lyase, polysaccharide-lyase-family 7, *Flavobacterium* sp. UMI-01, FlAlyA, recombinant alginate lyase

## Abstract

A major alginate lyase, FlAlyA, was purified from the periplasmic fraction of an alginate-assimilating bacterium, *Flavobacterium* sp. strain UMI-01. FlAlyA showed a single band of ~30 kDa on SDS-PAGE and exhibited the optimal temperature and pH at 55 °C and pH 7.7, respectively. Analyses for substrate preference and reaction products indicated that FlAlyA was an endolytic poly(mannuronate) lyase (EC 4.2.2.3). A gene fragment encoding the amino-acid sequence of 288 residues for FlAlyA was amplified by inverse PCR. The *N*-terminal region of 21 residues except for the initiation Met in the deduced sequence was predicted as the signal peptide and the following region of six residues was regarded as propeptide, while the *C*-terminal region of 260 residues was regarded as the polysaccharide-lyase-family-7-type catalytic domain. The entire coding region for FlAlyA was subjected to the pCold I—*Escherichia coli* BL21(DE3) expression system and ~eight times higher yield of recombinant FlAlyA (recFlAlyA) than that of native FlAlyA was achieved. The recFlAlyA recovered in the periplasmic fraction of *E. coli* had lost the signal peptide region along with the *N*-terminal 3 residues of propeptide region. This suggested that the signal peptide of FlAlyA could function in part in *E. coli*.

## 1. Introduction

Alginate is a heteropolysaccharide comprising β-d-mannuronic acid (M) and α-d-gluronic acid (G), which configure homopolymeric poly(M) and poly(G) blocks and heteropolymeric poly(MG) block in alginate polymer. Alginate occurs in intercellular matrices of brown seaweeds and biofilms of certain bacteria [1,2,3,4]. Due to its chemical stability, high viscosity and gel-forming property, alginate has been used for various applications, such as food additives, stabilizer, and gelling materials.

Alginate lyases (EC 4.2.2.3 and EC 4.2.2.11) are the enzymes that split glycosyl linkages of alginate chain via β-elimination mechanism producing unsaturated oligosaccharides, which possess an unsaturated sugar, 4-deoxy-l-erythro-hex-4-enopyranosyl-uronic acid, at the new non-reducing terminus [2,3,4]. This enzyme has been found to distribute over various organisms including bacteria, fungi, brown seaweeds, virus, and herbivorous mollusks [1,2,3,4], and they have been grouped under polysaccharide-lyase-family- (PL-) 5, 6, 7, 14, 15, 17, and 18 in the CAZy database [5]. Recently, degradation products of alginate produced by alginate lyases were found to exhibit a variety of bioactive functions, such as promotion of root growth in higher plants [6,7,8], acceleration of a growth rate of *Bifidobacterium* sp. [9], enhancing penicillin production in *Penicillium chrysogenum* [10], promotion of proliferation of macrophage, keratinocyte, and endothelial cells [11,12,13,14], and bringing down blood pressure in human [15,16]. In addition, alginate has been recognized as a promising carbohydrate source for biofuels. In reality, ethanol fermentation of alginate was recently succeeded by using genetically modified microorganisms that harbored alginate lyases and relating catabolic enzymes [17,18,19]. As alginate is abundant in brown seaweeds and there appear to be a large amount of brown seaweeds remained unused, the alginate will be a promising feedstock for useful biomaterials, including biofuels, in the future without causing food-fuel conflicts, which have emerged along with the bioethanol production from edible sugars.

To produce value-added materials from brown seaweeds, use of alginate lyases seems to be a key technique, as alginate lyase can directly degrade frond of brown seaweed releasing alginate oligosaccharides, and can convert elastic seaweed frond to slurry-like homogenates. The enzymatic degradation of alginate will expand the application areas of brown seaweeds by conferring novel properties. To realize degradation of seaweeds with alginate lyase in a practical scale, a large amount of enzyme should be provided in low cost. The promising sources for alginate lyase appeared to be the alginate-assimilating microbes that produce alginate lyases. There have been a number of reports on alginate-lyase producing bacteria, e.g., *Pseudomonas* sp., *Azotobacter* sp., *Alteromonas* sp., *Vibrio* sp., *Sphingomonas* sp., *Flavobacterium* sp.,* etc.* [4]. More than 500 of bacterial alginate-lyase genes have been enrolled in CAZy database [5]. These bacterial alginate lyases are usually secreted to periplasmic space or culture medium and extracellularly degrade alginate, while *Sphingomonas* sp. strain A1 are known to intracellularly degrade alginate that has been incorporated through the pits on cell membrane by the action of ABC transporter [4,20,21,22,23]. From the viewpoint of large-scale production of alginate lyases, it seems preferable to use secretory enzymes, since the extraction and purification of cytosolic enzymes are generally difficult and need labor.

To date, the authors’ group has been studying on alginate lyases from herbivorous marine mollusks, such as abalone, sea hare, and small snails [24,25,26,27,28,29]. These molluscan enzymes were the prominent members of polysaccharide PL-14 according to the searches for CAZy data base and available for the investigation of catalytic mechanism of PL-14 enzyme [27,29]. However, yields of these molluscan alginate lyases in *E. coli* expression systems were modest, although recombinant enzymes sufficient for biochemical studies, were successfully produced. On the other hand, the authors recently succeeded to isolate an alginate-assimilating bacterium, *Flavobacterium* sp. strain UMI-01, from seaweed litter. This strain appeared to secret a major alginate lyase, FlAlyA, to periplasmic space. Since the yield and specific activity of FlAlyA were considerably high and this enzyme could be produced in *E. coli* expression system, we focused on this enzyme as a candidate for practical applications.

In this paper, we report on the general properties of FlAlyA and high-yield expression of recombinant FlAlyA with an *E. coli* expression system.

## 2. Results

### 2.1. Characterization of Strain UMI-01

BLAST search for the nucleotide sequence of 16S ribosomal RNA gene of strain UMI-01 (DDBJ accession number, AB898090) to the Apollon database BA 6.0 indicated that this strain was an unreported *Flavobacterium* sp. The gene showing the highest sequence identity of 97% was of *F. omnivorum* strain AS1.2747 (accession number AF433174). The strain UMI-01 was rod-shaped (0.7–0.8 × 1.0–1.5 mm) and Gram-negative, and formed smooth, yellow, circular, convex, and entire edged colonies with a diameter of <1 mm on LB-agar plate. It grew aerobically and was capable of growing at 5–37 °C but incapable at 45 °C. It was positive in catalase, oxidase, and *p*-nitrophenyl-β-galactosidase, but negative in glucose oxidation, acid and gas productions, nitrate reduction, indole production, glucose oxidation, arginine dihydrolase, urease, gelatinase, cellulase, and flexirubin pigment production. It assimilated alginate, starch, d-mannose and d-mannitol but not *N*-acetyl-d-glucosamine, gluconic acid, *n*-capric acid, adipic acid, citric acid, and malic acid. The test with APIZYME (bioMerieux, Lyon, France) revealed that UMI-01 was positive in alkali phosphatase, Leu arylamidase, acid phosphatase and β-galactosidase. These properties supported the identification of strain UMI-01 as a *Flavobacterium* sp. Accordingly, we named this strain *Flavobacterium* sp. strain UMI-01.

### 2.2. Purification of FlAlyA

**Figure 1:**
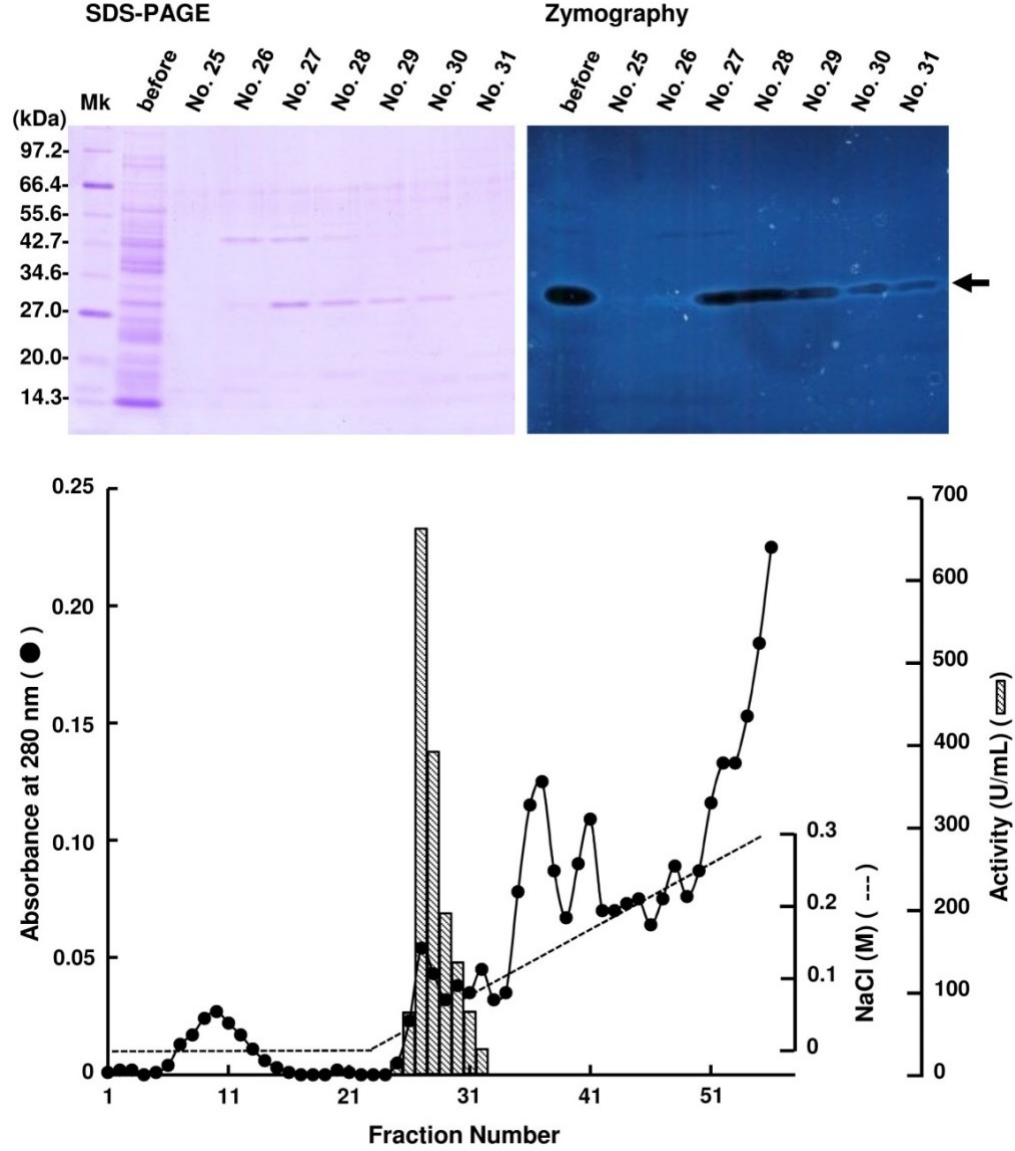
Purification of FlAlyA by TOYOPEARL DEAE-650M column chromatography. Proteins precipitated between 70% and 90% saturation of ammonium sulfate was subjected to the column of TOPYPEARL DEAE-650M (2.5 × 16 cm) and the proteins adsorbed to the column were eluted by the linear-gradient elution system from 0 to 0.3 M NaCl. One fraction contained 10 mL. SDS-PAGE and zymography for the fractions indicated with fraction numbers are shown in the top of the figure. An arrow indicates the position of FlAlyA.

**Figure 2:**
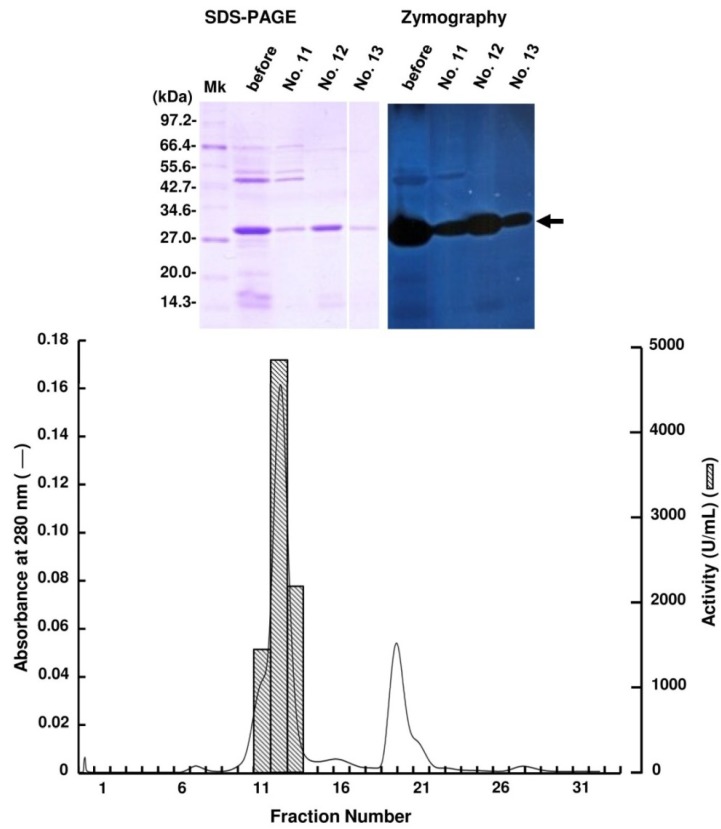
Purification of FlAlyA by gel-filtration through Superdex 75 10/300 GL. The FlAlyA fraction obtained in the TOYOPEARL DEAE-650M was dialyzed against 0.3 M NaCl—10 mM Tris-HCl (pH 7.6) and subjected to a Superdex 75 10/300 GL column (1 × 30 cm). One fraction contained 1 mL. SDS-PAGE and zymography for the fractions indicated with fraction numbers are shown in the top of the figure. An arrow indicates the position of FlAlyA.

Periplasmic fraction extracted from the strain UMI-01 was first subjected to ammonium sulfate fractionation. Proteins precipitated between 70% and 90% saturation of ammonium sulfate was collected by centrifugation at 10,000× *g* for 20 min, and dissolved in and dialyzed against 10 mM Tris-HCl (pH 7.6). Then, the dialysate was applied to a TOYOPEARL DEAE-650M column (2.5 × 16 cm) equilibrated with 10 mM Tris-HCl (pH 7.6) and eluted by the linear gradient from 0 to 0.3 M NaCl (Figure 1). Alginate lyase activity was detected in the fractions eluted at around 0.05–0.08 M NaCl. SDS-PAGE and zymography of these fractions indicated that the major alginate lyase was the 30 kDa protein. Thus, the fractions 27–29 were pooled and concentrated with a centrifugal concentrator VIVASPIN 20 (Sartorius, Gettingen, Germany), and subjected to AKTA FPLC equipped by Superdex 75 10/300 GL (GE Healthcare, Uppsala, Sweden) (Figure 2). Enzyme activity was detected in fractions 11–13, and SDS-PAGE and zymography indicated that the 30 kDa protein was the alginate lyase. Thus, we concluded that the 30 kDa protein was the major alginate lyase of strain UMI-01 and named it FlAlyA. The purification steps for FlAlyA from 5000 mL culture (OD600 nm = 1.5) are summarized in Table 1. Total 102,000 U of alginate lyase was extracted from the cells, and FlAlyA was purified 347.3-fold at a yield of 6.5% with the specific activity of 23,478 U/mg. More than 90% of FlAlyA appeared to be extracted in the periplasmic fraction since only 10% of activity was remained in the cytosolic fraction that was extracted by freeze and thaw, followed by sonication.

**Table 1:** Summary for purification of FlAlyA.

Purification Steps	Total Protein (mg)	Total Activity (U)	Specific Activity (U/mg)	Yield (%)	Purification (Fold)
Crude enzyme	1500.0	102,000	68	100.0	1.0
AS fractionation	61.0	29,646	486	29.3	7.2
DEAE-650M	0.8	9849	12,627	9.9	186.8
Purified FlAlyA	0.3	6574	23,478	6.5	347.3

### 2.3. Enzymatic Properties of FlAlyA

**Figure 3:**
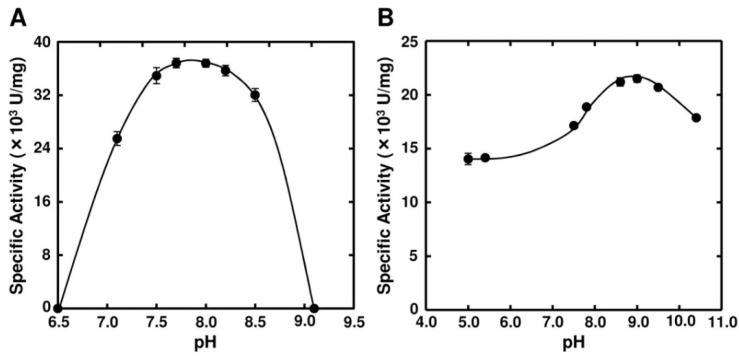
Effects of pH on the activity of FlAlyA. (**A**) pH dependence of FlAlyA. Activity was measured in the reaction mixtures adjusted to various pHs with 25 mM sodium phosphate buffer; (**B**) pH stability of FlAlyA. FlAlyA was pre-incubated in the buffers adjusted to various pHs at 30 °C for 3 h, then the residual activity was determined in the standard assay condition.

**Figure 4:**
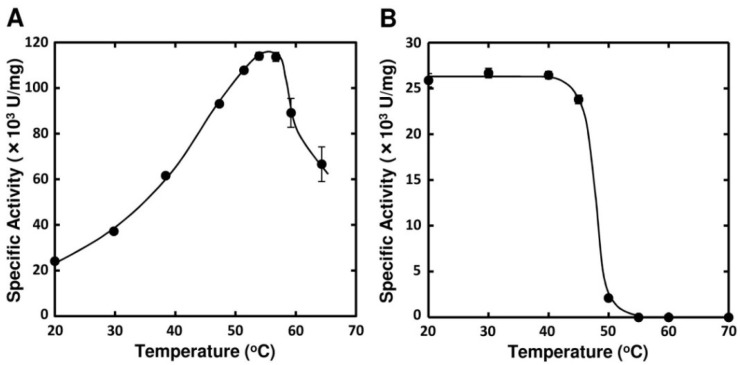
Effects of temperature on the activity FlAlyA. (**A**) Temperature dependence of FlAlyA. Activity was measured at various temperatures in the standard assay medium; (**B**) Heat stability of FlAlyA. FlAlyA was pre-incubated at various temperatures for 30 min and the residual activity was determined in the standard assay condition.

**Figure 5:**
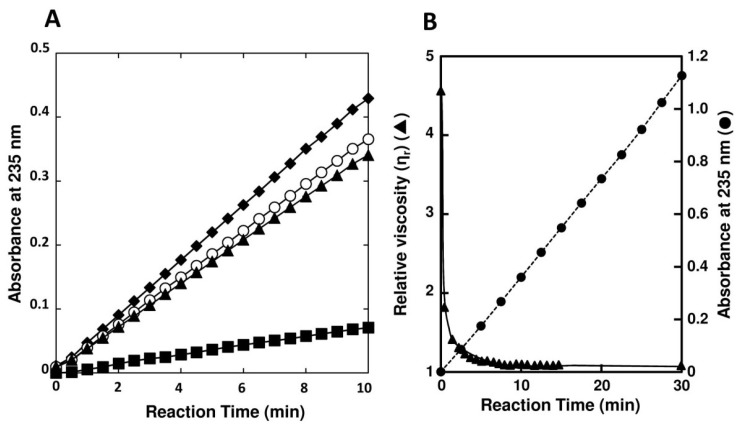
Substrate preference and endolytic action of FlAlyA. (**A**) Substrate preference of FlAlyA. Activity was measured in the reaction medium containing 0.12% (w/v) of sodium alginate (○), poly(M) block (♦), poly(MG) block (▲), or poly(G) block (■) at pH 7.0; (**B**) Decrease in viscosity of alginate by of FlAlyA. Viscosity of alginate (0.15% (w/v)) was measured with Ostwald’s viscometer at 30 °C and decrease in the viscosity by the addition of FlAlyA at final concentration of 0.003 mg/mL was continuously measured. The progress of degradation reaction by FlAlyA in the same condition was also recorded and shown in the same figure.

**Figure 6:**
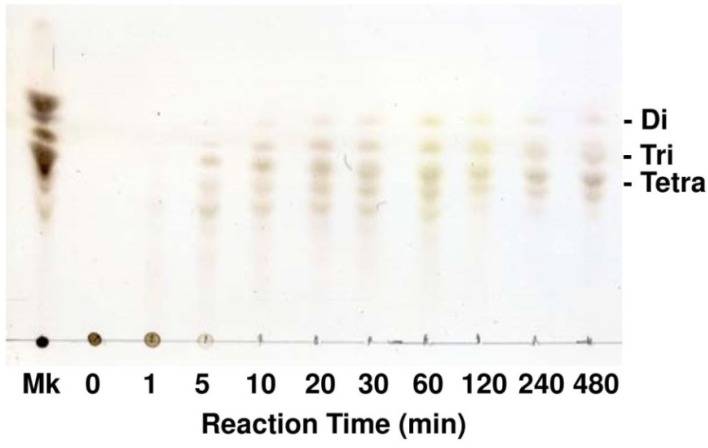
Analysis for the degradation products of alginate produced by FlAlyA. Alginate was degraded by FlAlyA and the degradation products were analyzed by TLC. Di, Tri, and Tetra indicate disaccharide, trisaccharide, and tetrasaccharide, respectively. Mk, degradation products of alginate produced by abalone crude enzyme [24,25]. The sizes of marker oligosaccharides were previously determined by BioGel P2 gel-filtration [24].

Optimal pH of FlAlyA was observed at around 7.8 and more than 80% of maximal activity was retained at pH 7.8–10.5 (Figure 3A,B). Optimal temperature of FlAlyA was observed at ~55 °C and the activity was not decreased by the incubation at 40 °C for 10 min (Figure 4A,B). Activity of FlAlyA was affected by several kinds of salts (Table 2). Addition of NaCl (100 mM) and MgCl_2_ (5 mM) to the reaction mixture increased the activity to ~30%, while CoCl_2_ (5 mM) and NiCl_2_ (5 mM) repressed the activity to ~50%. Metal chelators (5 mM) and disulfide-reducing reagents (5 mM) and other salts tested showed only modest effects. FlAlyA most preferably degraded poly(M) block, then sodium alginate, poly(MG) block and moderately poly(G) block (Figure 5A). Thus, FlAlyA was primarily identified as a poly(M) lyase (EC 4.2.2.3) possessing moderate activity toward poly(G) block. FlAlyA quickly decreased the viscosity of alginate in the early phase of reaction (Figure 5B), indicating that this enzyme degraded alginate with endolytic manner. The major degradation products of alginate produced by FlAlyA were di- and trisaccharide (Figure 6) similarly to other endolytic alginate lyases [3,4,24,25]. On the basis of these results, we concluded that FlAly was an endolytic poly(M) lyase (EC 4.2.2.3).

**Table 2:** Effects of salts and reagents on FlAlyA.

Substances	Concentration (mM)	Relative Activity (%)		
Non		100.0	±	2.0
NaCl	100	134.1	±	1.0
NaCl	5	106.6	±	0.5
KCl	5	108.8	±	1.0
CaCl_2_	5	118.8	±	0.6
MgCl_2_	5	126.0	±	0.9
CoCl_2_	5	57.1	±	2.3
MnCl_2_	5	101.1	±	0.7
NiCl_2_	5	47.1	±	1.0
EDTA	5	95.4	±	1.1
EGTA	5	106.4	±	0.9
Dithiothreitol	5	93.2	±	0.6
2-Mercaptoethanol	5	92.6	±	0.5

### 2.4. Analysis for Primary Structure of FlAlyA

**Figure 7:**
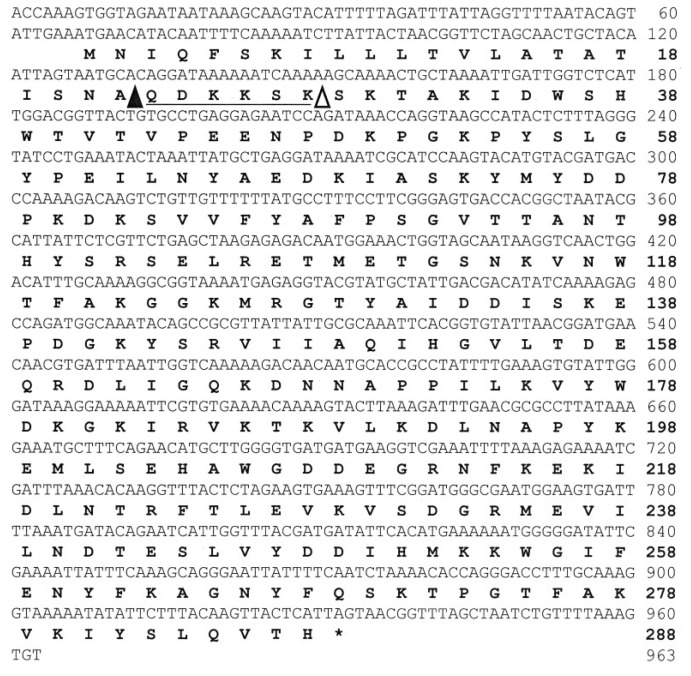
The nucleotide and deduced amino-acid sequences of FlAlyA. The end of signal peptide predicted by SignalP 4.0 server [30] is indicated with a closed triangle (▲) in the amino-acid sequence, while the actual *N*-terminus of native FlAlyA is indicated with an open triangle (∆). The putative propeptide region is indicated by an underline.

The *N*-terminal amino-acid sequence of FlAlyA was determined by protein sequencer as SKTAKIDWSHWTVTVPEENPDKPGKPYSLGYPEILNYA-. This sequence showed the amino-acid identity higher than 50% with those of known PL-7 enzymes, suggesting that FlAlyA is a member of PL-7. Therefore, we synthesized two degenerated forward primers, FlAly-1F and FlAly-2F, on the basis of the partial amino-acid sequences of FlAlyA, SKTAKIDW (1st–8th residues) and WSHWTVTVP (8th–16th residues), respectively, and a degenerated reverse primer, PL7-Rv, on the basis of an amino-acid sequence, YFKAGNY, which is highly conserved among PL-7 enzymes [4] (Table 3). Using these degenerated primers and UMI-01 genome DNA as a template, we performed nested PCR as follows: The first PCR was carried out with the primer set FlAly-1F and PL7-Rv, and the second PCR was with FlAly-2F and PL7-Rv, and a DNA fragment of approximately 400 bp (FlAly-1-DNA) was successfully amplified. Next, inverse PCR was performed with specific primers,* i.e.*, InvFw1, InvFw2, InvRv1, and InvRv2, which were synthesized on the basis of the nucleotide sequence of FlAly-1-DNA (Table 3), and a DNA fragment of approximately 3245 bp (FlAly-Inv-DNA) was amplified. Finally, a DNA fragment comprising 963 bp (FlAlyA-DNA) that covered an entire coding region of FlAlyA was amplified from FlAly-Inv-DNA with a specific primer pair, FlAlyA-Fw and FlAlyA-Rv. The nucleotide and the deduced amino-acid sequence of FlAlyA-DNA are shown in Figure 7. These sequences are available from DNA Data Bank of Japan with the accession number AB898059. Analysis for the deduced amino-acid sequence of 288 residues of FlAlyA by SignalP software version 4.0 [30] indicated that the *N*-terminal region of 21 residues except for the initiation Met was a signal peptide for secretion. On the other hand, the *N*-terminus of native FlAlyA was the 29th Ser. This indicated that the region QDKKSK (23rd–28th residues in the deduced sequence) was removed from FlAlyA during maturation. Thus, this region was considered to correspond to the propeptide of FlAlyA. This region may modify the translocation efficiency of FlAlyA to periplasmic space and/or localize the catalytic domain to periplasmic space. This was suggested in the experiment for the expression of recombinant FlAlyA in *E. coli* (see the following section).

**Table 3:** Primers used for amplification of FlAlyA gene.

Primer Name	Nucleotide Sequence (Corresponding Amino-Acid Sequence)
FlAly-1F	5′-WSNRACNGCNAARATHGAYTGG-3′
	(SKTAKIDW)
FlAly-2F	5′-TGGWSNCAYTGGACNGTNACNGTNCC-3′
	(WSHWTVTVP)
PL7-Rv	5′-TANARNCCNGCYTTRAARTA-3′
	(YFKAGNY)
InvFw1	5′-AATGAGAGGTACGTATGCTATTGACGAC-3′
InvFw2	5′-GCGTTATTATTGCGCAAATTCACGG-3′
InvRv1	5′-CAACAGACTTGTCTTTTGGGTCATCGTA-3′
InvRv2	5′-GGATGCGATTTTATCCTCAGCATAATTT-3′
FlAlyA-Fw	5′-ACCAAAGTGGTAGAATAATAAAA-3′
FlAlyA-Rv	5′-ACACTTTAAAACAGATTAGCTAAACCG-3′

Amino-acid sequences used for designing of primers are shown in the parentheses. N, adenine or guanine or cytosine or thymine; R, adenine or guanine; Y, cytosine or thymine; H, adenine or cytosine or thymine; S, cytosine or guanine; and W, adenine or thymine.

### 2.5. Expression of Recombinant FlAlyA in E. coli

To examine if recombinant FlAlyA (recFlAlyA) can be produced at a certain yield in *E. coli* and secreted to the periplasmic fraction by the aid of the signal peptide of FlAlyA, entire coding region of FlAlyA,* i.e.*, spanning from the initiation Met to termination codon, was subjected to the pCold I—*E. coli* BL21 (DE3) expression system. Expression of recFlAlyA was induced by the addition of 0.1 mM IPTG and concomitant decrease in the cultivation temperature to 15 °C. After the 24 h cultivation, bacterial cells were harvested by centrifugation at 4600× *g* for 20 min. The bacterial pellets were gently suspended in the osmotic stressing buffer (30 mM Tris-HCl (pH 8.0), 20% (w/v) sucrose and 1 mM EDTA) with a spatula and placed on ice for 1 h with occasional suspension. The suspension was then centrifuged at 6000× *g* for 20 min obtaining supernatant. This extraction was repeated once more and the two supernatant was used as periplasmic fraction of *E. coli*. The cell residues were then suspended with 10 mM sodium phosphate (pH 7.0) and frozen at −20 °C and thawed at room temperature, and homogenized by sonication. The homogenate was centrifuged at 12,000× *g* for 15 min and the soluble fraction was used as a cytosolic fraction. The recFlAlyA in the periplasmic and cytosolic fractions were separately purified by Ni-NTA affinity chromatography. As shown in Table 4, the ratio for the yields of recFlAlyA in the periplasmic and cytosolic fractions was approximately 1:17. Total yield of recFlAlyA was 84,272 units from 500 mL culture. This was approximately eight times higher than the yield of native FlAlyA,* i.e.*, 102,000 units from 5000 mL culture (see Table 1). Overall properties of recFlAlyA from the two fractions were comparable to those of native FlAlyA (data not shown). However, the recFlAlyA was found to consist of plural species with different *N*-terminal structures. Thus, the *N*-terminal amino-acid sequences determined with specimens blotted to PVDF membrane indicated that recFlAlyA from the periplasmic fraction had lost the signal peptide region along with the *N*-terminal three residues,* i.e.*, QDK, of the propeptide region (see Figure 7). On the other hand, the *N*-terminal sequence of cytosolic recFlAlyA comprised of two sequences (two amino acids were determined in each sequencing cycle). By the sequence analysis, we concluded that one recombinant possessed intact signal peptide and another had lost the signal peptide but retained the propeptide. The ratio for the former to latter was estimated to be ~1:3 from the yields of PTH-amino acids in the sequence chromatograph. These results suggested that the signal peptide of FlAlyA functioned in part in *E. coli* as a secretion signal; however, the further cleavage of propeptide region was necessary for the easy release of recFlAlyA from periplasmic space. In other words, the propeptide region may affect the translocation of FlAlyA to periplasmic space and/or anchor FlAlyA to structural materials in periplasmic space.

**Table 4:** Recovery of recFlAlyA in periplasmic and cytosolic fractions of *E. coli*.

Fractions	Total Protein (mg)	Total Activity (U)	Activity Yield (%)
Periplasmic	12.35	4688	5.6
Cytosolic	75.45	79,584	94.4
Sum of fractions		84,272	100.0

## 3. Discussion

Seaweeds play important ecological roles in ocean providing comfortable habitat environments and diets for marine organisms [31,32,33]. Seaweeds have also been recognized as important bioresources for human as foods, food additives, industrial materials, biofuels,* etc.* Seaweeds contain a number of specific storage and structural polysaccharides and sugar derivatives [34,35]. Among the seaweed’s polysaccharides, alginate from brown seaweeds appears to be the most potential carbohydrate because of its high productivity and biomass. To date, attempts have been made to produce “value-added” materials such as functional oligosaccharides [8,9,10,11,12,13,14,15,16] and bioethanol [17,18,19] from brown seaweeds. In the production of such materials, degradation of alginate seems to be a prerequisite process. Acid-hydrolysis of alginate is known to be inefficient because of the formation of elastic and fibrous aggregates by acids. Hydrolysis of alginate in high concentrations of strong acid, e.g., 50%~80% (v/v) sulfuric acid, results low yield of hydrolysates and produces large amount of dehydrated toxic by-products. Neutralization of acid hydrolysates, which will result a large amount of salt wastes, is also a knotty problem to be solved. On the other hand, degradation of alginate by alginate lyase is obviously superior to acid-hydrolysis because this enzyme efficiently degrades alginate under non-acidic conditions producing no toxic by-product. Further, a monosaccharide unit of alginate, e.g., 4-deoxy-l-erythro-5-hexoseulose uronic acid (DEH), which was recently shown to be available for ethanol fermentation [17,18,19], can be produced by oligoalginate lyase (exolytic alginate lyase) [25,36]. Such potentiality of alginate lyase has led us to exploit novel and suitable enzymes that can be used for practical applications. In the present study, we isolated *Flavobacterium* sp. strain UMI-01 from seaweed litter, characterized its major alginate lyase FlAlyA, and achieved high yield expression in *E. coli*.

FlAlyA was considered to be secreted in the periplasmic space of strain UMI-01 since it was easily released from cell pellets by the osmotic stressing. Approximately 90% of total activity was recovered in this fraction. The molecular mass of FlAlyA was estimated to be 30 kDa by SDS-PAGE, which was well consistent with the molecular mass of 29856.8 Da that was calculated from the deduced amino-acid sequence. FlAlyA showed optimal temperature and pH at 55 °C and pH 7.7, respectively. FlAlyA efficiently degraded poly(M) block, sodium alginate and poly(MG) blocks in this order, but moderately poly(G) block, and quickly decreased the viscosity of sodium alginate in the early phase of reaction. On the basis of these properties, we concluded that FlAlyA was an endolytic poly(M) lyase (EC 4.2.2.3) showing moderate activity toward poly(G) block.

**Figure 8:**
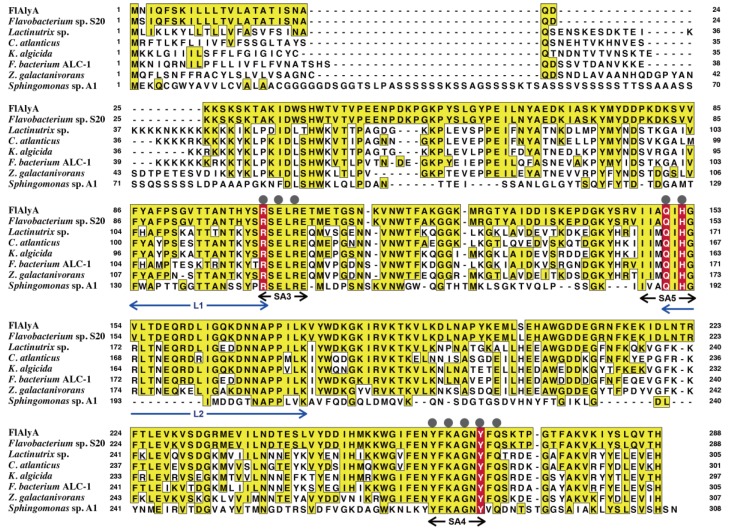
Comparison of amino-acid sequences of PL-7 alginate lyases. FlAlyA, *Flavobacterium* sp. UMI-01 alginate lyase FlAlyA; *Flavobacterium* sp. S20, *Flavobacterium* sp. S20 alginate lyase Alg2A (GenBank accession number AEB69783) (Huang* et al.*, 2013); *Lacinutrix* sp. 5H-3-4-7, *Lacinutrix* sp. 5H-3-7-4 alginate lyase (GenBank accession number YP004580029) (Klippel* et al.*, 2011); C. Atlanticus HTCC2559, Croceibacter atlanticus HTCC2559 hypothetical protein CA2559_11483 (GenBank accession number YP003717038) (Oh* et al.*, 2010); K. Algicida, Kordia Algicida hypothetical protein KAOT1_04210 (GenBank accession number ZP02164186) (Lee* et al.*, 2011); F. Bacterium ALC-1, Flavobacteriales bacterium ALC-1 alginate lyase (GenBank accession number ZP02182531); Z. galactanivorans, Zobellia Galactanivorans aly2A gene product (GenBank accession number YP004737047); *Sphingomonas* sp. A1, *Sphingomonas* sp. A1 alginate lyase A1-II’ (GenBank accession number BAD16656) (Miyake* et al.*, 2004). The amino-acid residues identical to those of the FlAlyA sequence are indicated by painting with yellow. The regions highly conserved in PL-7 alginate lyases,* i.e.*, SA3, SA4, and SA5 [37], are indicated with black arrows. Catalytic residues conserved in PL-7 enzymes [37,38,39,40] are highlighted with red letters. Residues located on the surface of active cleft of PL-7 enzymes [40] are indicated with gray circles. Two lid loops (L1 and L2) covering active cleft of *Sphingomonas* sp. A1-II’ [37,38] are indicated with blue arrows.

The amino-acid sequence of FlAlyA was deduced from the gene fragment amplified by inverse PCR. In the *N*-terminus of the deduced sequence, a signal peptide region of 21 residues and a propeptide region of 6 residues were identified, while the *C*-terminal region of 260 residues was identified as the catalytic domain of FlAlyA. This domain shared the amino-acid identity of 99% to the corresponding sequence of alginate lyase Alg2A from *Flavobacterium* sp. S20 [23] and ~50% identities to other PL-7 alginate lyases (Figure 8). Thus, FlAlyA was regarded as a PL-7 enzyme most closely related to Alg2A. Three-dimensional structures of PL-7 alginate lyases have been solved with some bacterial enzymes [37,38,39,40]. The regions highly conserved in PL-7 alginate lyases,* i.e.*, SA3, SA4, and SA5 [37,38,39], were also well conserved in FlAlyA (Figure 8). In addition, catalytic residues of PL-7 enzymes and the residues located on the surface of active cleft were highly conserved in FlAlyA. While, two lid loops (L1 and L2) covering active cleft of *Sphingomonas* sp. A1-II’ [37,38] were not conserved in FlAlyA. These loops were suggested to oscillate flexibly upon accommodation of substrates and relate to the broad substrate specificity to A1-II’ [38]. Difference in the substrate preference between A1-II’ and FlAlyA,* i.e.*, multiple preference and poly(M)-block preference, respectively, may be ascribable to the differences in the higher order structures of these loop regions. General properties of FlAlyA were similar to those of Alg2A and the primary structures of FlAlyA and Alg2A were almost identical (99% identity). However, substrate preferences of two enzymes were apparently different from each other. Namely, FlAlyA was regarded as poly(M) lyase while Alg2A was identified as poly(G) lyase [23]. The reason for the difference in substrate preferences between FlAlyA and Alg2A is still obscure; however, the assay conditions for alginate lyase activity are known to affect the substrate preferences of the PL-7 enzymes. We recently noticed that FlAlyA became capable of degrading poly(G) block in lower pH conditions. The details for the substrate preference of FlAlyA should be further investigated from the structural aspect. Three-dimensional structure analysis for FlAlyA is now under way.

Recombinant FlAlyA (recFlAlyA) was found to be produced at ~eight times higher yield by the pCold I—*E. coli* expression system than the enzyme from native strain UMI-01. It was also found that a part of recFlAlyA was readily extracted in the periplasmic fraction by the osmotic stressing. This enzyme,* i.e.*, periplasmic recFlAlyA, had lost the signal peptide and the *N*-terminal three residues of propeptide,* i.e.*, QDK of QDKKSK. On the other hand, the recFlAlyA extracted by freeze and thaw followed by sonication after the osmotic stressing,* i.e.*, cytosolic recFlAlyA, possessed both signal and propeptide regions or only propeptide region. These results suggested that the signal peptide of FlAlyA could function in *E. coli* as a secretion signal; however, partial cleavage of propeptide region was also necessary for facile release of FlAlyA. Thus, the propeptide region was considered to relate to the translocation and/or anchoring of the enzyme to some structural matrices in periplasmic space. In case of secretion of native FlAlyA by UMI-01, the enzyme may be secreted by the aid of signal peptide to periplasmic region and further processed by cleavage of the propeptide region, and then released to the periplasmic space as the matured FlAlyA. To improve the yield of recFlAlyA in the *E. coli* expression systems, manipulation, and modification of the propeptide region may be important. In this respect, it should be examined whether or not the manipulation of propeptide region improves the yield of recFlAlyA in the periplasmic fraction of *E. coli*.

## 4. Experimental Section

### 4.1. Materials

TOYOPEARL DEAE-650M was purchased from Toyo soda Mfg, Co. (Tokyo, Japan), and Superdex 75 10/300 GL was from GE Healthcare UK Ltd. (Little Chalfont, Bucking Hamshire, England). Sodium alginate (*Macrocystis pyrifera* origin) was purchased from Sigma-Aldrich (St. Louis, MO, USA). Poly(M) and poly(MG) and poly(G) blocks of alginate were prepared by the limited acid-hydrolysis of alginate according to the method of Gacesa and Wusteman [41]. Briefly, 50 g of sodium alginate was suspended in 1 L of 0.3 N HCl and partially hydrolyzed at 100 °C for 20 min. Poly(MG) block released to the supernatant was collected by centrifugation, neutralized, and precipitated with ethanol in a final concentration of 60%. Insoluble materials after the partial hydrolysis were again hydrolyzed with 0.3 N HCl for 24 h and collected by centrifugation. The precipitates were suspended in 500 mL of distilled water, dissolved by the addition of small amount of 1 M NaOH, and adjusted to pH 2.85 with 1 N HCl. The precipitates formed (poly(G) block) were collected by centrifugation, dissolved in 10 mM Na_2_CO_3_, and precipitated with ethanol. While the supernatant (poly(M) block) was neutralized with 0.1 M Na_2_CO_3_ and precipitated with ethanol. By this procedure, 4.6 g, 17.9 g, and 12.4 g of poly(MG), poly(M) and poly(G) blocks, respectively, were obtained. According to the circular dichroism analysis [42], mannuronate content in the poly(M) block and poly(MG) block were estimated to be 86% and 64%, respectively, and gluronate content in the poly(G) block was 99%. While, mannuronate content in the original alginate was 60%. TLC-60 plates were purchased from Merck KGaA (Dermstadt, Germany). Bacto Trypton and Yeast Extract were purchased from Becton and Dickinson (Sparks, MD, USA). The other chemicals were reagent grade from Wako Pure Chemical Industries Ltd. (Osaka, Japan).

### 4.2. Isolation of Strain UMI-01

An alginate-assimilating bacterium strain UMI-01 was isolated from seaweed litter as follows. The litter, comprising mainly brown seaweed *Coccophora langsdorfii*, was collected in seashore of Otaru, Hokkaido, Japan in June 2010. Two grams of the litter were added to 50 mL of minimum salt medium containing 1% (w/v) alginate (alginate-MS medium; 1% (w/v) sodium alginate, 0.38% (w/v) Na_2_HPO_4_, 0.27% (w/v) KH_2_PO_4_, 0.036% (w/v) NH_4_Cl, 0.02% (w/v) MgCl_2_, and 0.1% (v/v) of trace element solution (0.97% (w/v) FeCl_3_, 0.78% (w/v) CaCl_2_, 0.02% (w/v) CoCl_2_ 6H_2_O, CuSO_4_ 5H_2_O, 0.01% (w/v) NiCl_3_ 6H_2_O and 0.01% CrCl_3_ 6H_2_O in 0.1 N HCl)). The medium was first incubated at 20 °C for a week and then 1 mL of the culture was transferred to 100 mL of alginate-MS medium. The culture was incubated under aerobic conditions in a temperature controlled shaker BR-43FL (TAITEC, Tokyo, Japan) at 30 °C and 120 rpm for 24 h and aliquots of the culture were streaked with a platinum loop on the 1.5% (w/v) agar plates containing alginate-MS medium (alginate-agar-MS plate). The bacterial colonies formed on the plates by the cultivation at 30 °C for five days were separately picked up and repeatedly cultivated on alginate-agar-MS plate until isolation completed. By these procedures, 18 independent bacterial isolates, strains UMI-01–UMI-18, were obtained (“UMI” was named after the authors’ research program “Universal Marine Industry for Green Innovation” supported by Ministry of Education, Culture, Sports, Science, and Technology, Japan). We selected the strain UMI-01 as the most promising alginate-lyase producing bacterium since the growth rate of this strain was the highest among the isolates. Identification of bacterial species on the basis of nucleotide sequence of 16S rRNA gene and the physiological and biochemical properties was carried out in TechnoSuruga Laboratory Co., Ltd. (Shimizu, Shizuoka, Japan). The results indicated that the strain UMI-01 was a bacterium belonging to *Flavobacterium* (detailed characteristics of this strain will be described under “Results”).

### 4.3. Preparation of Periplasmic Fraction from Strain UMI-01

Single colony of strain UMI-01 was inoculated to 5 mL of alginate-MS medium and cultivated at 30 °C and 120 rpm for 24 h. The culture was then transferred to 500 mL of alginate-MS medium in a baffled-bottom 3 L flask and cultivated at 30 °C and 100 rpm for 24 h (OD 600 nm reached ~1.5). For one preparation of enzyme, total 5 L of culture was used. Cells were harvested from the 5 L culture by the centrifugation with Hitachi Himac centrifuge (CR 20 G) at 4 °C and 4600× *g* for 30 min. The periplasmic fraction was then extracted from the cell pellets with 500 mL of a buffer containing 30 mM Tris-HCl (pH 8.0), 20% (w/v) sucrose and 1 mM EDTA (TSE buffer; Quan* et al.* [43]) at 0 °C for 1 h with occasional suspension with a spatula. The suspension was then centrifuged at 6000× *g* and 4 °C for 20 min obtaining the supernatant as a periplasmic fraction. The extraction was repeated again and the two supernatant fractions were pooled and used for the purification of FlAlyA.

### 4.4. Assay for Alginate Lyase Activity

Alginate lyase activity was assayed at 30 °C in a standard reaction mixture containing 10 mM sodium phosphate buffer (pH 7.0), 0.12% (w/v) of sodium alginate, poly(M), poly(MG), or poly(G) block substrates, and 0.005–0.05 mg/mL enzyme. Progress of reaction was monitored by measuring absorbance at 235 nm of the reaction mixture with a spectrophotometer model U-3010 (HITACHI, Tokyo, Japan) equipped by a thermal controlling unit SP-12R (TAITEC). One unit (U) of alginate lyase was defined as the amount of enzyme that increases Abs235 nm to 0.01 for 1 min. To assess the pH dependence of FlAlyA, activity was assayed in mixtures adjusted to pH 5.9–10.5 with 25 mM sodium phosphate buffer. pH stability was assessed by measuring the activity remaining after the incubation at 30 °C for 3 h in various pH media. To assess temperature dependence, the activity was measured at 20–65 °C in the standard reaction mixture. Heat stability was assessed by measuring activity after the incubation at various temperatures for 30 min. The average values of triplicate measurements were indicated along with a standard deviation.

### 4.5. Thin Layer Chromatography for Degradation Products of Alginate

A total of 5 mg/mL of alginate dissolved in 10 mM sodium phosphate buffer (pH 7.0) was degraded with 50 U/mL of FlAlyA at 30 °C. At appropriate time intervals, aliquots were withdrawn and heated at 100 °C for 2 min to inactivate enzyme. Two microliters of reaction products were then applied to a TLC-60 plate and developed with a solvent comprising 1-butanol, acetic acid and water (2:1:1, v:v:v). The products separated on the plate were stained by spraying 10% (v/v) sulfuric acid in ethanol followed by heating at 110 °C for 10 min.

### 4.6. SDS-PAGE and Zymography

SDS-PAGE was carried out with 10% polyacrylamide slab gel (10 cm × 10 cm × 0.1 cm) according to the method of Porzio and Pearson [44]. After the electrophoresis, proteins were stained with 0.15% (w/v) Coomassie Brilliant Blue R-250 in 50% (v/v) methanol—10% acetic acid, and background of the gel was destained with 15% (v/v) acetic acid—5% (v/v) methanol. Zymography for alginate lyase was carried out with the SDS-PAGE gel containing 0.12% sodium alginate as a substrate. After the electrophoresis at 4 °C, the gel was soaked in 25 mL of 10 mM sodium phosphate (pH 7.0) containing 20% (v/v) isopropanol and gently agitated at 4 °C for 30 min to remove SDS. The gel was then equilibrated with 50 mL of 10 mM sodium phosphate (pH 7.0) at 4 °C for 30 min and further incubated at 30 °C for 6 h. Degradation of alginate in the gel was detected as a clear halo against opaque background by the immersion of 10% (w/v) cetylpyridinium chloride aqueous solution.

### 4.7. Determination of Protein Concentration

Protein concentration was determined by the method of Lowry* et al.* [45] using bovine serum albumin fraction V as a standard protein.

### 4.8. Determination of N-Terminal Amino-Acid Sequence

The *N*-terminal amino-acid sequence of FlAlyA was determined with an ABI Procise 492 protein sequencer (Applied Biosystems, Foster City, CA, USA) using specimens electrically blotted to polyvinylidene difluoride (PVDF) membrane (Applied Biosystems, Foster City, CA, USA).

### 4.9. Cloning and Sequencing of FlAlyA Gene

Total DNA (genomic DNA) of strain UMI-01 was prepared with an ISOHAIR kit (Nippon Gene, Tokyo, Japan). Genomic PCR was performed using Phusion DNA polymerase (New England Biolabs, Ipswich, MA, USA) in 20 μL of reaction mixture containing 50 mM KCl, 10 mM Tris-HCl (pH 8.3), 1.5 mM MgCl_2_, 0.125 mM each of dATP, dTTP, dGTP, and dCTP, and 4 pmol primers, 50 ng template DNA, and 0.5 units of the DNA polymerase under the following conditions: 98 °C for 2 min, 30 cycles of 95 °C for 30 s, 55 °C for 30 s, and 72 °C for 1 min. Inverse PCR was performed using a self-circularized *Kpn* I-digested genomic DNA as a template in the same conditions as described above except for 5-min extension reaction was performed in the end of PCR. The amplified DNA was ligated to pTAC-1 vector (BioDynamics, Tokyo, Japan) with an A-attachment Mix (Toyobo, Osaka, Japan) and cloned with *E. coli* DH5α. Nucleotide sequences were determined with a BigDye-Terminator Cycle Sequence kit (Applied Biosystems, Foster City, CA, USA) and a DNA sequencer 3130 × L (Applied Biosystems, Foster City, CA, USA).

### 4.10. Expression of Recombinant FlAlyA in Escherichia coli

A DNA fragment covering the entire coding region of FlAlyA was amplified from the FlAlyA-DNA by the PCR with a specific primer set. The amplified DNA was ligated to pCold I (Takara, Shiga, Japan), which had been digested with *Nco* I and *Bam* HI, by homologous recombination using In-Fusion system (Clontech Laboratories, Mountain View, CA, USA). To attach the 8× Gly8× His-tag to the *C*-terminus of recombinant protein, the pCold I vector had been modified as follows. (1) The original translation enhancing element, 6× His-tag sequence and Factor Xa site were eliminated. (2) The DNA sequence encoding GSGGGGGGGGHHHHHHHH (8× Gly8× His) was added to the 3′-end of translational region in frame with *BamH* I sequence. (3) The *Nde* I site was changed to *Nco* I site to adopt its internal ATG sequence as the translational initiation codon. The recombinant pCold I was then introduced to *E. coli* BL21(DE3) and the recombinant *E. coli* was cultivated in 500 mL of 2 x YT medium containing 0.05 mg/mL ampicillin at 37 °C 12 h. The expression of recombinant FlAlyA (recFlAlyA) was induced by the addition of 0.1 mM IPTG followed by cooling to 15 °C. The periplasmic fraction of the *E. coli* was prepared by the osmotic stressing method as described for the native strain UMI-01. The cytosolic recFlAlyA, which remained un-extracted in *E. coli* after the osmotic stressing, was then extracted by the freeze and thaw followed by sonication with an Ultrasonic Homogenizer VP-050 (TAITEC, Tokyo, Japan) at 20 kHz and 30 W for total 1 min (15 s × 4 times with each 1 min interval). These recFlAlyA were purified by affinity chromatography with Ni-NTA resin (Qiagen, Tokyo, Japan) and dialyzed against 10 mM sodium phosphate buffer (pH 7.0) before use.

## 5. Conclusions

Alginate from brown seaweeds has been recognized as a promising marine biomass that can be used for the production of functional oligosaccharides and fermentable sugars. In this study, a PL-7-type endolytic alginate-degrading enzyme FlAlyA was isolated from *Flavobacterium* sp. strain UMI-01 and successfully expressed as a recombinant enzyme by using an *E. coli* expression system. Since acid hydrolysis of alginate requires considerable energy costs and brings significant amount of salt wastes upon neutralization, enzymatic degradation is obviously superior way for depolymerization of alginate in view of the Green Chemistry concept. Since FlAlyA was found to be easily expressed with an *E. coli* system, the recombinant FlAlyA will be a firm base for the biotechnological studies of alginate lyases. Structure/function studies on FlAlyA using mutagenesis techniques are now underway. 
